# Effect of Geographical Indication Information on Consumer Acceptability of Cooked Aromatic Rice

**DOI:** 10.3390/foods9121843

**Published:** 2020-12-11

**Authors:** Sara E. Jarma Arroyo, Victoria Hogan, Debra Ahrent Wisdom, Karen A. K. Moldenhauer, Han-Seok Seo

**Affiliations:** 1 Department of Food Science, University of Arkansas, Fayetteville, AR 72704, USA; sejarmaa@uark.edu (S.E.J.A.); vjhogan@uark.edu (V.H.); 2 Division of Agriculture Rice Research and Extension Center, University of Arkansas Systems, Stuttgart, AR 72160, USA; dahrent@uark.edu (D.A.W.); kmolden@uark.edu (K.A.K.M.)

**Keywords:** aromatic rice, geographical indication, origin, label, consumer, sensory, rice

## Abstract

Geographical indication (GI) labeling is used to represent information about specific geographical origins of target products. This study aimed at determining the impact of GI information on sensory perception and acceptance of cooked aromatic rice samples. Ninety-nine participants evaluated cooked rice samples prepared using each of three aromatic rice varieties both with and without being provided with GI information. Participants rated the acceptance and intensity of the cooked rice samples in terms of appearance, aroma, flavor, texture, and overall liking, and also reported how important the GI information was to them. The results showed that consumers rated the cooked rice samples higher in appearance and overall liking when provided with GI information. Interestingly, participants who valued “state-of-origin” information more highly exhibited increased hedonic ratings of cooked rice samples when provided with GI information, but not when no GI information was given. Participants provided with GI information rated flavor or sweetness intensities of cooked aromatic rice samples closer to just-about-right than those without such information. This study provides empirical evidence about how GI information modulates sensory perception and acceptance of cooked aromatic rice samples. The findings will help rice industry, farmers, and traders better employ GI labeling to increase consumer acceptability of their rice products.

## 1. Introduction

Intrinsic cues (e.g., sensory properties of appearance, texture, aroma, and taste) of products often work together with extrinsic cues (e.g., packaging, brand name, food origin, and labeling), thereby influencing what types of food consumers choose [1,2,3]. For example, when consumers are exposed to both packaging information and sensory properties (e.g., appearance or flavor) of food products, their food choice is influenced by both factors, not just how much they like the product based on its sensory qualities [1,2]. This trend has been observed in a variety of test samples, including beef [4], yogurt [5], apple juice [6], blackcurrant squashes [7], sweeteners [8], and mixed vegetable juice [2]. In a recent study by Samant and Seo [2], bitterness intensity and brand liking were found to play an important role in purchase-related behavior with respect to mixed vegetable juice products. In particular, previous studies have highlighted substantial impacts of brand or labeling on consumer liking, preference, and purchase-related behaviors toward food samples [2,6,9,10,11]. In a study by Stolzenbach et al. [6], when consumers were shown the brand of apple juice they were tasting, their liking scores were impacted and changed compared to when they did a blind tasting of the same juice. Similarly, Torres-Mereno et al. [9] found that liking and preference toward chocolate were different when consumers were given the same samples with or without label information. Notably, the label effect was found to be more pronounced when consumers better understood information about the label [12,13] or when they gave more value to the label attribute [10].

Country-of-origin labeling on imported food products is mandated by the U.S. Customs and Border Protection [14]. Furthermore, the United States Department of Agriculture (USDA) also requires some domestic products, including meat, produce, and some nuts, to include origin labeling [15]. This has led to a large amount of research focused on how country-of-origin labeling impacts consumers’ perception of and willingness to buy different food items. Consumers have been found to demonstrate a higher preference for products where the country-of-origin is domestic rather than foreign [16,17]. For example, many individuals were found to not only prefer, but also be willing to pay more for, domestic beef than imported beef [18,19]. This finding extends to other foods as well, including organic produce [20], seafood [21], dairy [22], genetically modified foods [23], and rice [24,25]. For example, Lee et al. [24] found that Korean consumers were willing to pay more for rice when they knew that it had been grown in their own country. In a study conducted in Japan, including country-of-origin labeling on rice increased the value of domestic rice and decreased the value of imported rice [25]. Banović et al. [21] found that, in seafood, a country-of-origin label is more important to consumers than are health and nutrition claims.

While most origin labeling focuses on “country”, some foods are given more specific identifying information such as the “state” or “region” within the country where they were produced. This is often referred to as geographical indication (GI) labeling. Menapace et al. [26] found that, in the case of olive oil, many consumers were willing to pay a higher price for products with a GI label that associated certain specific locations with higher-quality products. Including a GI label on a product seems to make the most difference to consumers in terms of a willingness to pay a higher price when purchasing produce and other agricultural products [27]. Such preferences can be highly shaped by government legislation that can help promote the purchase of locally-grown agricultural products [28]. Furthermore, educating consumers on how agriculture products are grown and the benefits of consuming local produce is another way of helping increase consumption of local foods [29].

Aromatic rice is a good example of an agricultural product in which the market in some countries is strongly dominated by imported varieties. For example, most U.S. rice imports of aromatic varieties come primarily from Thailand (Jasmine), followed by India and Pakistan (Basmati). The amount of Jasmine rice imported from Thailand increased by over 250,000 tons between 2010 and 2019 [30]. Thailand Jasmine rice has also won the title of “World’s Best Rice” five out of the past ten years at the World Rice Conference [31]. These factors reflect a growing demand for Jasmine rice and an increase in its favorable perception. As a result, U.S. rice breeders have been working for a number of years to create domestic varieties of Jasmine; this has led to a couple of varieties being successfully developed [32,33]. However, Suwansri et al. [34] found that when Asian consumers in the U.S. evaluated both domestic and imported Jasmine rice, they demonstrated preference for imported Jasmine even when they did not know the origin of the samples. None of the previous studies, however, have accounted for how geographical indication information modulates sensory perception of cooked rice, raising the question of how inclusion of geographical indication labels might help increase consumer acceptance of domestic aromatic rice varieties.

Building on previous findings asserting that geographical indication labels played a crucial role in consumer perception and acceptance of food products [16,17,18,19,20,21], this study aimed to determine how geographical indication information affects sensory perception and acceptance of cooked aromatic rice samples among U.S. consumers, Northwest Arkansas residents in particular.

## 2. Materials and Methods

### 2.1. Rice Samples

To determine whether GI information influences consumer perceptions of aromatic rice at both state and country levels, two U.S aromatic rice cultivars and one imported aromatic rice sample were selected for this study. “ARoma 17” (ARV) and “Jazzman-2” (LAV) are Jasmine-type aromatic rice cultivars grown in the United States. ARoma 17 was developed at the University of Arkansas System Division of Agriculture’s Rice Research and Extension Center [35] and Jazzman-2 was developed at the Louisiana State University Agricultural Center [33]. Since Jasmine rice imports to the U.S. come primarily from Thailand [30], a Jasmine sample of Thailand origin was selected for comparison. The Thailand Jasmine sample (Vin Sanh Trading Corporation, City of Industry, CA, USA) (THV) from an unknown cultivar was obtained from a local Asian market (Northwest Arkansas, AR, USA).

The three un-cooked aromatic rice samples used in this study differed in their physicochemical properties: moisture content, amylose content, pasting properties, crude protein, surface lipid content, and surface color (Table S1). To determine moisture content, amylose content, and pasting properties of milled rice, a 60-g portion from one head rice sub-sample was ground into flour using a cyclone mill (3010–30, UDY, Fort Collins, CO, USA) with a 0.5 mm screen. Moisture content of milled-rice samples was measured according to the American Association of Cereal Chemists (AACC) method 44–15.02 [36]. Amylose content was determined by the simplified iodine-assay method [37]. Pasting properties of the rice samples were determined using a Rapid Visco Analyser (RVA Super 4, Newport Scientific, Warriewood, Australia). Crude protein content, surface lipid content, and surface color properties, i.e., L* (light vs. dark), a* (red vs. green), and b* (yellow vs. blue), of milled rice samples were determined by scanning approximately 60 g of head-rice kernels using NIR spectroscopy (NIR-DA 7200, Perten Instruments, Huddinge, Sweden). All analyses were performed in triplicate.

### 2.2. Preparation of Cooked Rice Samples

Three hundred grams of milled rice of each rice variety were cooked in an electric rice cooker (RC3314W rice cooker, Black & Decker, Beachwood, OH, USA) with a 1:1.8 rice-to-water (*w*/*w*) ratio. The optimum cooking duration for each rice variety was determined by the Ranghino test for milled rice [38]. After the samples had been cooked, a plastic spoon was used to softly fluff and mix them three times in the rice cooker to ensure homogeneity. Thirty grams of cooked rice were then spooned into a three-digit coded 118 mL Styrofoam cup (Dart Container Corporation, Mason, MI, USA) and allowed to cool down to 70 °C. The cups were then covered with airtight lids and presented to the participants.

Descriptive sensory analysis conducted at the University of Arkansas Sensory Science Center (Fayetteville, AR, USA) revealed that the cooked rice samples of the three aromatic rice varieties differed in terms of sensory attributes (Table S2). More specifically, five professionally-trained panelists who had completed at least 50 h of descriptive analysis panel training with a wide range of different products, including cooked rice, evaluated the three cooked rice samples in terms of 30 attributes (4 appearances, 6 aromas, 10 flavors, 3 basic tastes, 7 textural properties) on scales ranging from 0 (not at all) to 15 (very strong). Prior to sample evaluation, orientation and training sessions conforming to the “Rice Aromatic Scale” methodology employed by Jarma Arroyo and Seo [39] were conducted for twelve hours on four different days. Table S3 lists the definitions and reference intensities of individual appearance, flavor, and texture-related attributes that were evaluated. Samples were presented to the panelists in a sequential monadic fashion, with 10-min breaks between sample presentations. During each break, spring water (Clear Mountain Spring Water, Taylor Distributing, Heber Springs, AR, USA) was provided as a palate cleanser. The entire evaluation was repeated on three separated days to provide three replicated sensory analyses of the cooked rice samples.

### 2.3. Consumer Acceptance Test of Cooked Rice Samples

This study was conducted per the Declaration of Helsinki for studies on human participants. The protocol used in this study was approved by the Institutional Review Board of the University of Arkansas (Fayetteville, AR, USA). Prior to participation, a written informed consent was obtained from each participant.

Ninety-nine participants (72 females and 27 males) with a mean age of 46 years (standard deviation (SD) = 14) were recruited through a consumer profile database provided by the University of Arkansas Sensory Science Center. The sample size was within the range of 40–100 consumers, as recommended by Gacula and Rutenbeck [40] for consumer testing. A majority of participants were Caucasians (*n* = 83), followed by African Americans (*n* = 7), Hispanics (*n* = 4), American Indians (*n* = 3), and others (*n* = 2). All had also self-reported eating cooked rice at least twice a month.

Prior to sample presentation, a verbal introduction about the experimental protocol was given to each participant. Instructions and scales were presented using sensory analysis software, Compusense Cloud^®^ (Compusense Inc., Guelph, ON, Canada). Following the orientation session, each participant was presented with four cooked rice samples (one warm-up and three test samples) and a white plastic spoon, in a sequential monadic fashion consistent with the Williams Latin Square design [41].

**Figure 1:**
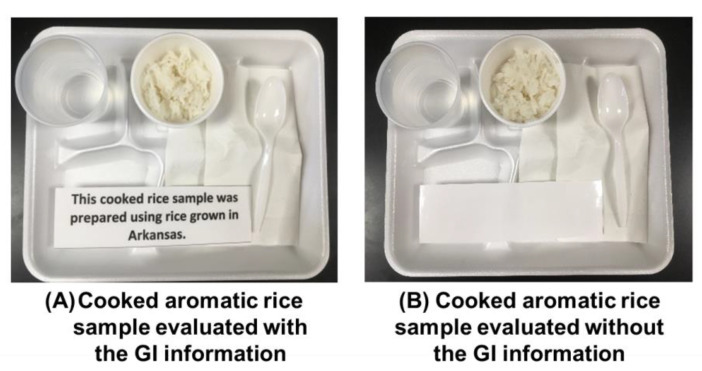
Examples of cooked aromatic rice samples evaluated with or without geographical indication (GI) information. (**A**) Cooked aromatic rice samples were evaluated in the presence of GI information: this cooked rice sample was prepared using rice grown in (Arkansas, Louisiana, or Thailand). (**B**) Cooked aromatic rice samples were also evaluated in the absence of GI information; a blank rectangular card containing no information was presented.

All participants underwent two experimental sessions: a “GI labeling” session and a “no GI labeling” session. More specifically, during the GI labeling session participants were presented with a rectangular white card next to the rice samples that indicated where the rice samples had been grown, e.g., “This cooked rice sample was prepared using rice grown in (Arkansas, Louisiana, or Thailand)”, as shown in Figure 1A. Since (1) this study was targeted to determine how Arkansas residents might respond to cooked rice samples in the presence of GI information showing either the country-of-origin or the state-of-origin, and (2) the LAV sample was originally grown in the state of Louisiana, labeling of Louisiana was presented for the LAV sample. In contrast, during the no GI labeling session, participants were provided cooked rice samples placed next to a blank rectangular card of the same dimensions as the one used for GI labeling but containing no information about the sample (Figure 1B). The sessions were separated by one week and their order was counterbalanced across participants to eliminate any confounding order effects [42].

Participants were asked to evaluate the cooked rice samples with respect to color, firmness, stickiness, chewiness, aroma, flavor, saltiness, sweetness, and bitterness on 5-point Just-About-Right (JAR) scales (1 = much too little, 3 = JAR, and 5 = much too much). In addition, likings of appearance, aroma, flavor, texture, and overall impression of each cooked rice sample were rated on 9-point hedonic scales ranging from 1 (dislike extremely) to 9 (like extremely), respectively. Finally, free text comments describing aspects participants liked and disliked about the samples were gathered. A 90-s break was allowed between sample presentations, with spring water (Clear Mountain Spring Water, Taylor Distributing, Heber Springs, AR, USA) presented as a palate cleanser.

Since consumers have been shown to demonstrate implicit attitudes toward information about food products origin [43,44,45,46], participants were asked to report how important the country-of-origin (COO) or state-of-origin (SOO) information was to them when consuming aromatic rice, using 9-point scales ranging from 1 (extremely unimportant) to 9 (extremely important).

### 2.4. Statistical Analysis

Statistical analyses were performed using SPSS 26.0 for Windows^TM^ (IBM SPSS Inc., Chicago, IL, USA) and XLSTAT software (Addinsoft, Long Island, NY, USA). Physicochemical data was analyzed using a one-way analysis of variance (ANOVA), treating “rice sample” as a fixed effect. Descriptive sensory data were analyzed using a three-way ANOVA, treating “rice sample”, “panelist”, and “repetition” as fixed effects, along with their two-way interactions. A statistically-significant difference was defined to exist when *p* < 0.05. The results of the physicochemical and descriptive sensory analyses are shown in Supplementary Materials (Tables S1 and S2) because they are not within the main scope of this study.

To determine the global effect of GI information on consumer acceptance of cooked aromatic rice, a two-way repeated measures multivariate analysis of variance (RM-MANOVA) was performed using “rice sample” and “labeling condition” as fixed effects. If the sphericity assumption was found to be violated via the Mauchly’s sphericity test, the degrees of freedom were adjusted using a “Greenhouse-Geisser” correction. If a significant effect was indicated by the RM-MANOVA, further univariate RM-ANOVAs were conducted. When a significant difference in means was indicated by the RM-ANOVA, post hoc comparisons between the test samples were performed using Bonferroni *t*-tests. A partial eta-squared (*η_p_*^2^) value was used to measure effect size for ANOVA. *η_p_*^2^ values of 0.01, 0.06, and 0.14, respectively, were considered to be small, medium, and large effect sizes [47,48].

Partial correlation analyses were used to examine relationships between the importance levels of “country-of-origin” (COO) and hedonic ratings of attributes for cooked aromatic rice, with controlling the importance levels of “state-of-origin” (SOO) as a covariate. For the importance levels of “state-of-origin”, partial correlation analyses were conducted the other way around.

Free response data from the comments (what participants liked or disliked) were analyzed using text exploration and chi-square tests. Text exploration is a technique that allows processing and analyzing of semi-structured and unstructured textual data [49]. Groups of words with similar roots or meaning (e.g., aroma and smell) were clustered into a single term (e.g., aroma). A chi-square test was then conducted to determine whether the frequency of a specific term, reported as to what participants liked or disliked, differed between the two GI information conditions. To help visualize the GI information effect on the comments regarding what participants liked or disliked, a “word cloud” was generated by JMP Pro software (version 16, SAS Institute, Cary, NC, USA).

The absolute delta (|Δ|) JAR scores were computed by subtracting each JAR rating from the just-about-right value (i.e., 3). As conducted for analyzing the hedonic ratings, a two-way RM-MANOVA was performed treating “rice sample” and “labeling condition” as fixed effects for the |Δ|JAR scores. If a significant effect was indicated by the RM-MANOVA, univariate RM-ANOVAs were also conducted. In addition, using a chi-square test, the frequency of JAR selection for each JAR attribute per sample was compared between the two GI information conditions. A statistically-significant difference was defined as when *p* < 0.05.

## 3. Results

### 3.1. Effect of GI Information on Hedonic Impression of Cooked Aromatic Rice Samples

The two-way RM-MANOVA revealed no significant interaction between “rice sample” and “GI information” on hedonic ratings of cooked aromatic rice samples (*p* = 0.70). Additionally, univariate RM-ANOVAs found no significant interactions on the ratings of overall liking (*p* = 0.22), appearance (*p* = 0.06), aroma (*p* = 0.87), flavor (*p* = 0.29), and texture (*p* = 0.17) in cooked aromatic rice samples.

**Figure 2:**
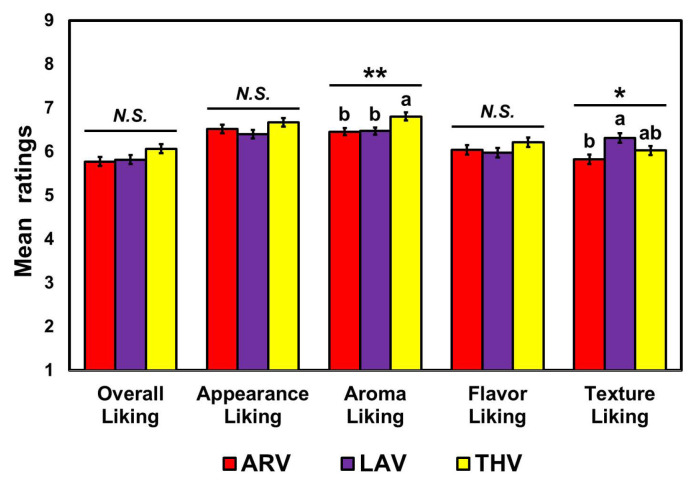
Comparisons among the three cooked aromatic rice samples: ARoma 17 (ARV), Jazzman-2 (LAV), and Thailand Jasmine (THV), with respect to hedonic ratings of attributes and overall impression. * and ** represent a significant difference at *p* < 0.05 and *p* < 0.01, respectively. Different letters with mean ratings within a category indicate a significant difference at *p* < 0.05. *N.S.* represents no significant difference at *p* < 0.05.

RM-MANOVA revealed significant effects of “rice sample” (Wilks’ lambda = 0.68, *p* < 0.001, *η_p_*^2^ = 0.32) and “GI information” (Wilks’ lambda = 0.84, *p* = 0.005, *η_p_*^2^ = 0.16) on hedonic ratings of cooked aromatic rice samples. Further univariate RM-ANOVAs revealed that cooked rice samples of three varieties were found to differ significantly in terms of aroma liking (*p* = 0.004, *η_p_*^2^ = 0.06) and texture liking (*p* = 0.02, *η_p_*^2^ = 0.04) (Figure 2). Post hoc pairwise comparisons revealed that the imported Thailand Jasmine (THV) cooked rice was rated as having a more pleasant aroma than cooked rice samples of domestic varieties: ARV (*p* = 0.02) and LAV (*p* = 0.02). With respect to texture of cooked rice, the LAV was better-liked than the ARV (*p* = 0.02). No significant sample differences were found in the hedonic ratings of overall liking (*p* = 0.19), appearance (*p* = 0.16), and flavor (*p* = 0.27) of cooked rice samples.

**Figure 3:**
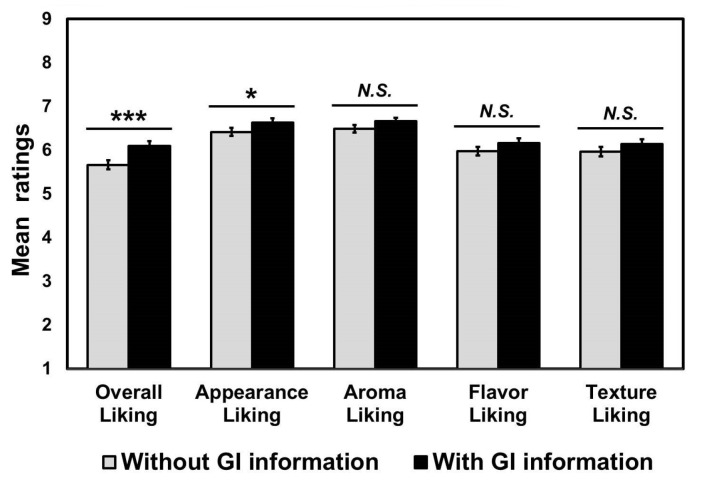
Comparisons between the two geographical indication (GI) information conditions with respect to hedonic ratings of attributes and overall impression across the three cooked aromatic rice samples. * and *** represent a significant difference at *p* < 0.05 and *p* < 0.001, respectively. Different letters with mean ratings within a category indicate a significant difference at *p* < 0.05. *N.S.* represents no significant difference at *p* < 0.05.

Univariate RM-ANOVAs also revealed significant effects of “GI information” on ratings of overall liking (*p* < 0.001, *η_p_*^2^ = 0.11) and appearance liking (*p* = 0.04, *η_p_*^2^ = 0.04) of cooked rice samples (Figure 3). Specifically, participants liked cooked rice samples with GI information given, more than when it was missing. However, no significant effects of GI information were found in the hedonic ratings of aroma (*p* = 0.07), flavor (*p* = 0.14), and texture (*p* = 0.18) of the cooked rice samples.

Participants rated importance of country-of-origin (COO) or state-of-origin (SOO) information when consuming aromatic rice products. Table 1 shows how the COO or SOO scores were related to hedonic ratings of cooked rice samples for each variety. Interestingly, while no significant correlations were found between the COO scores and hedonic ratings of cooked rice samples when no GI information was provided, significant correlations were observed when GI information was provided. More specifically, participants who considered the GI information about “country-of-origin” (COO) to be less important gave higher ratings of overall liking (*r_p_* = −0.21, *p* = 0.04), appearance liking (*r_p_* = −0.23, *p* = 0.02), and flavor liking (*r_p_* = −0.23, *p* = 0.02) to the ARV sample when GI information was present. Similarly, they gave higher ratings of texture liking (*r_p_* = −0.24, *p* = 0.02) for the LAV sample and aroma liking (*r_p_* = −0.20, *p* = 0.049) for the THV sample, respectively, when GI information was present. Contrary patterns for the GI information condition were observed in the relationships between the SOO scores and hedonic ratings of cooked rice samples. As shown in Table 1, participants who considered the GI information about “state-of-origin” (SOO) to be more important gave higher ratings of overall liking (*r_p_* = 0.23, *p* = 0.02), appearance liking (*r_p_* = 0.25, *p* = 0.01), flavor liking (*r_p_* = 0.25, *p* = 0.01), and texture liking (*r_p_* = 0.25, *p* = 0.01) to the ARV sample when GI information was present. They also gave higher ratings of texture liking (*r_p_* = 0.24, *p* = 0.02) for the LAV sample and aroma liking (*r_p_* = 0.27, *p* = 0.008) for the THV sample, respectively, in the GI information condition.

### 3.2. Effect of GI Information on Positive or Negative Comments Regarding Cooked Aromatic Rice Samples

**Figure 4:**
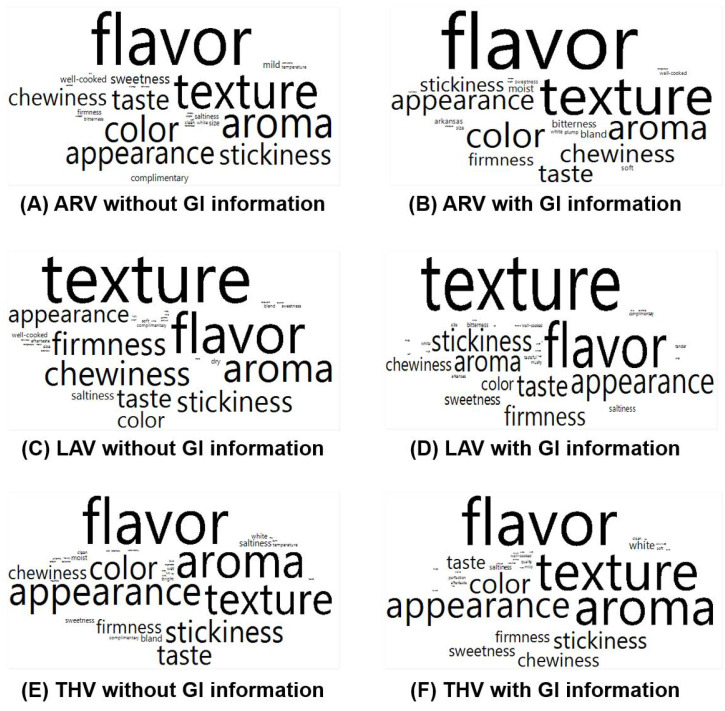
Geographical indication (GI) information-induced variations in the relative percentages of positive terms for each of the three cooked aromatic rice samples: (**A**,**B**) ARoma 17 (ARV), (**C**,**D**) Jazzman-2 (LAV), and (**E**,**F**) Jasmine from Thailand (THV). The size of a term in the visualization represents the number of responses reported as what participants liked for each of the three cooked aromatic rice samples. The layout of each term has no specific meaning. A list of terms reported by at least 10 participants for each cooked aromatic rice sample is shown in Table S4.

Participants commented on what they liked and disliked, respectively, for each cooked aromatic rice sample. As shown in Figure 4, there were no significant differences between the two GI information conditions with respect to frequency of specific positive terms for each cooked aromatic rice sample (for all, *p* > 0.05). While “flavor” was most frequently commented on as being liked for the ARV or THV sample, “texture” was most often commented on for the LAV sample, independent of the GI information condition. A list of terms reported by at least 10 consumer participants with respect to what they liked for each of the three cooked aromatic rice samples is shown in Table S4.

**Figure 5:**
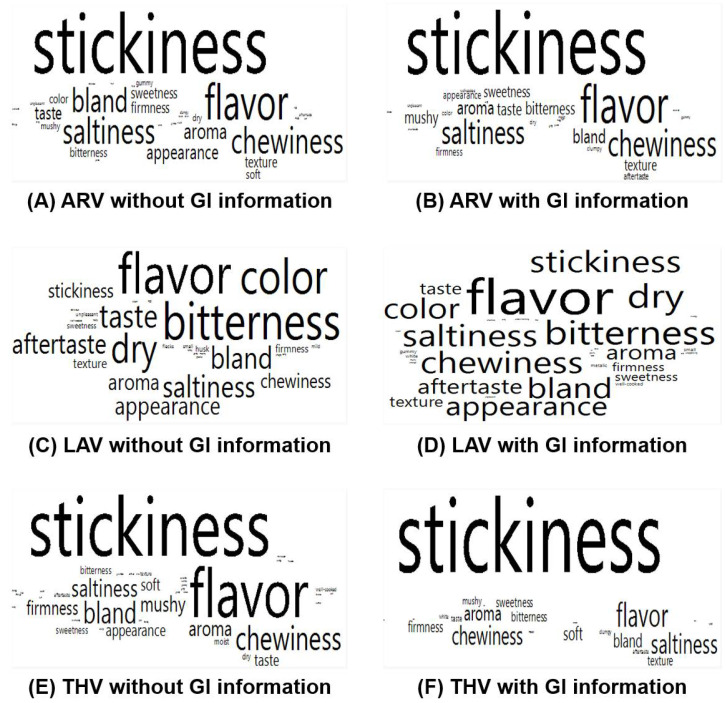
Geographical indication (GI) information-induced variations in the relative percentages of negative terms for each of the three cooked aromatic rice samples: (**A**,**B**) ARoma 17 (ARV), (**C**,**D**) Jazzman-2 (LAV), and (**E**,**F**) Jasmine from Thailand (THV). The size of a term in the visualization represents the number of responses reported as what participants liked for each of the three cooked aromatic rice sample. The layout of each term has no specific meaning. A list of terms reported by at least 10 participants for each cooked aromatic rice sample is shown in Table S5.

Figure 5 shows negative comments on each of the three cooked aromatic rice samples under the two GI information conditions. Among the three cooked rice samples, while “stickiness” was most often commented on as being disliked for the ARV or THV sample, “flavor” was the most frequently commented on for the LAV sample, independent of the GI information condition. There were no significant differences between the two GI information conditions with respect to frequency of specific negative terms directed toward each cooked aromatic rice sample (for all, *p* > 0.05), except for “stickiness” for the THV sample. More specifically, for the THV sample, “stickiness” was most frequently designated as a negative attribute when GI information was given (*n* = 46) than when it was absent (*n* = 29) (*p* = 0.01). A list of terms reported by at least 10 consumer participants with respect to what they disliked for each of the three cooked aromatic rice samples is given in Table S5.

### 3.3. Effect of GI Information on the Just-About-Right (JAR) Ratings of Cooked Aromatic Rice Samples

A two-way RM-MANOVA revealed no significant interaction between “rice sample” and “GI information” on the absolute delta JAR scores (|Δ|JAR), i.e., the absolute differences between individual JAR scores and the just-about-right score (3), of cooked aromatic rice samples (*p* = 0.20). Further univariate RM-ANOVAs revealed no significant interactions on |Δ|JAR scores of individual attributes (for all, *p* > 0.05), except for color (*p* = 0.009) and sweet taste (*p* = 0.03). While no differences between the GI information conditions were observed in the |Δ|JAR scores of color or sweetness for the ARV or THV sample, the color or sweetness intensities of the LAV sample were closer to the JAR score when the GI information was given compared to when it was absent. In a similar vein, percentages of JAR scores with respect to color (*p* = 0.003) or sweetness (*p* = 0.046) in the LAV sample were significantly higher in the presence of GI information than in the no-information condition (Table 2).

**Figure 6:**
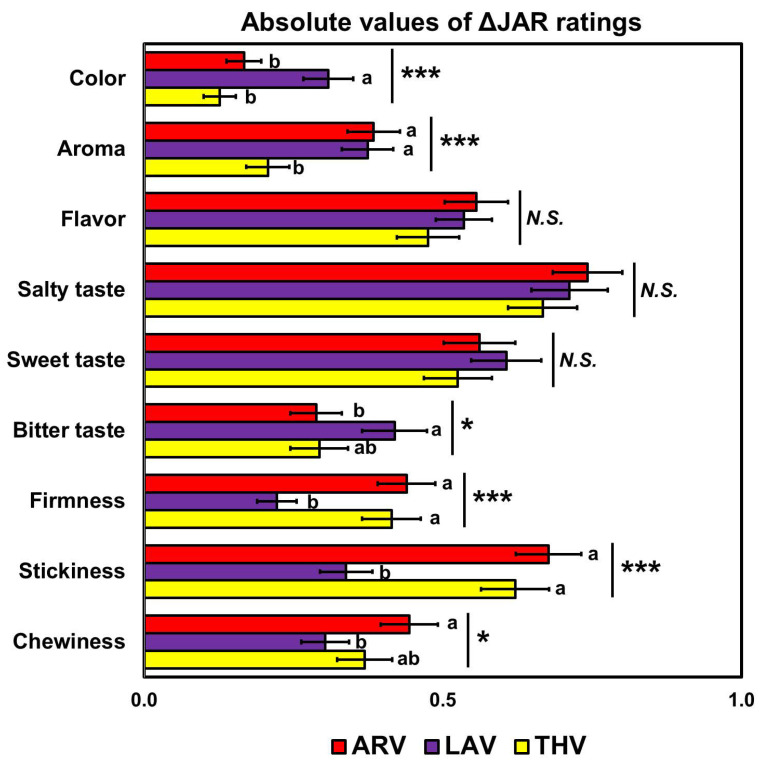
Comparisons among the three cooked aromatic rice samples: ARoma 17 (ARV), Jazzman-2 (LAV), and Thailand Jasmine (THV), with respect to absolute delta of Just-About-Right (JAR) ratings. The absolute delta (|Δ|) JAR scores were computed by subtracting each JAR rating from the JAR value (i.e., 3). * and *** represent a significant difference at *p* < 0.05 and *p* < 0.001, respectively. Different letters with mean ratings within a category indicate a significant difference at *p* < 0.05. *N.S.* represents no significant difference at *p* < 0.05.

The two-way RM-MANOVA revealed a significant main effect of “rice sample” (Wilks’ lambda = 0.45, *p* < 0.001, *η_p_*^2^ = 0.55) on the |Δ|JAR scores of cooked aromatic rice samples. Univariate RM-ANOVAs further revealed that the |Δ|JAR scores of the three varieties differed significantly with respect to color (*p* < 0.001, *η_p_*^2^ = 0.11), aroma (*p* < 0.001, *η_p_*^2^ = 0.08), bitter taste (*p* = 0.02, *η_p_*^2^ = 0.04), firmness (*p* < 0.001, *η_p_*^2^ = 0.09), stickiness (*p* < 0.001, *η_p_*^2^ = 0.12), and chewiness (*p* = 0.04, *η_p_*^2^ = 0.03) (Figure 6). Participants rated surface color of the ARV (*p* < 0.001) or THV (*p* < 0.001) samples closer to their JAR scores (i.e., lower |Δ| scores) than those of the LAV sample. They also rated aroma intensities of the THV sample closer to their JAR scores than those of the ARV (*p* < 0.001) or LAV (*p* = 0.001) sample. The participants also rated bitterness intensities of the ARV sample closer to their JAR scores than those of the LAV sample (*p* = 0.04). In contrast, they rated firmness intensity of the LAV sample closer to their JAR scores than those of the ARV (*p* < 0.001) or THV (*p* = 0.005) sample. Similarly, the stickiness intensities of the LAV sample were closer to the JAR scores than those of the ARV (*p* < 0.001) or THV (*p* = 0.001) sample. The chewiness intensities of the LAV sample were also closer to the JAR scores than those of the ARV sample (*p* = 0.047). No significant effects of “rice sample” were observed on the |Δ|JAR scores for flavor (*p* = 0.39), salty taste (*p* = 0.32), and sweet taste (*p* = 0.23). These trends were also observed in the percentages of JAR with respect to color, aroma, firmness, stickiness, or chewiness (Table 2). For example, for the aroma attribute rated in either GI information condition, significantly more participants rated the THV sample to have JAR intensity when compared to the ARV or LAV sample (for all, *p* < 0.05).

**Figure 7:**
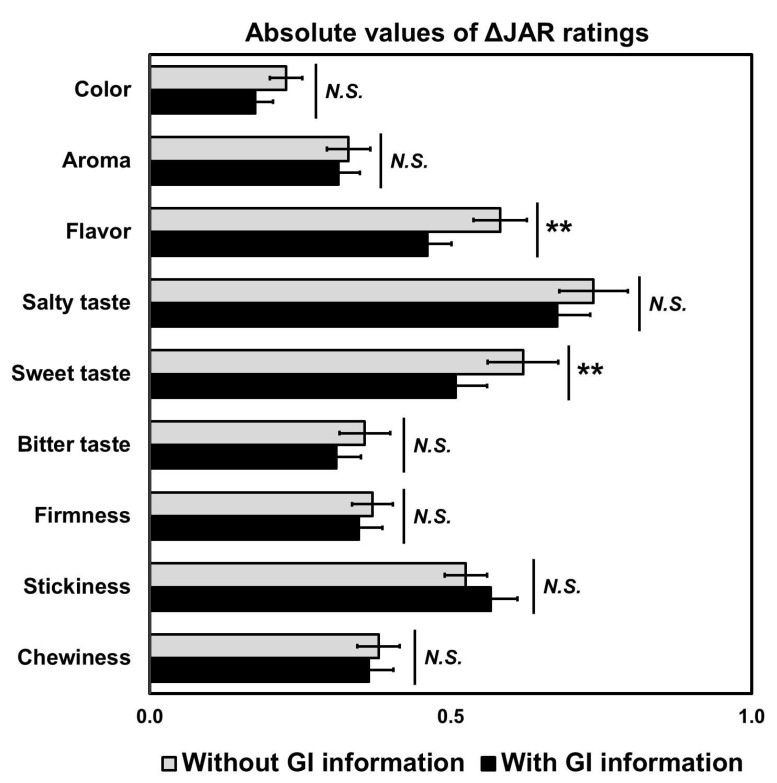
Comparisons between the two geographical indication (GI) information conditions with respect to absolute delta of Just-About-Right (JAR) ratings. The absolute delta (|Δ|) JAR scores were computed by subtracting each JAR rating from the just-about-right value (i.e., 3). ** represents a significant difference at *p* < 0.01. Different letters with mean ratings within a category indicate a significant difference at *p* < 0.05. *N.S.* represents no significant difference at *p* < 0.05.

While the two-way RM-MANOVA revealed no significant effect of “GI information” (Wilks’ lambda = 0.86, *p* = 0.11, *η_p_*^2^ = 0.14) on the |Δ|JAR scores of cooked aromatic rice samples, univariate RM-ANOVAs showed significant effects on the |Δ|JAR scores with respect to flavor (*p* = 0.004, *η_p_*^2^ = 0.08) and sweet taste (*p* = 0.004, *η_p_*^2^ = 0.08) (Figure 7). No significant effects of “GI information” were observed on the |Δ|JAR scores of color (*p* = 0.08), aroma (*p* = 0.64), salty taste (*p* = 0.12), bitter taste (*p* = 0.14), firmness (*p* = 0.56), stickiness (*p* = 0.37), and chewiness (*p* = 0.69).

## 4. Discussion

### 4.1. Impact of Geographical Indication Information on Consumer Acceptance of Cooked Aromatic Rice Samples

This study shows how geographical indication (GI) information modulates consumer perception and acceptance of cooked aromatic rice samples. As seen in Figure 3, participants liked cooked aromatic rice samples with GI information significantly more than those without GI information. In particular, the effect of GI information inclusion was statistically detectable in the appearance liking, although mean hedonic ratings of other attributes were also slightly higher in the presence of GI information than in its absence. It should be noted that the surface color of cooked rice has been found to be the most important factor in determining acceptances of Jasmine rice products among Asian consumers in the U.S. [34]. This finding, therefore, seems to some extent be applied also to non-Asian consumers in the U.S. (there were no Asian participants in this study). In our additional analysis using multiple linear regression (adjusted *R*^2^ = 0.77, *p* < 0.001), appearance liking (standardized coefficient *β* = 0.09, *t* = 2.55, *p* = 0.01) was found as one of the significant contributors to overall liking of cooked aromatic rice, along with flavor liking (standardized coefficient *β* = 0.55, *t* = 15.22, *p* < 0.001) and texture liking (standardized coefficient *β* = 0.39, *t* = 9.39, *p* < 0.001), when GI information was provided. However, in the multiple linear regression (adjusted *R*^2^ = 0.76, *p* < 0.001) without GI information provided, appearance liking (*p* = 0.89) was not found to contribute to predicting overall liking; only flavor liking (standardized coefficient *β* = 0.58, *t* = 17.20, *p* < 0.001) and texture liking (standardized coefficient *β* = 0.41, *t* = 12.04, *p* < 0.001) were found to be significant contributors. This result suggests that geographical indication information can lead consumers to like the appearance aspects of cooked aromatic rice samples, thereby increasing their preference for the samples with such GI information.

Our result related to the significant effect of GI information on overall liking was consistent with previous findings where origin information was found to exert a significant increase in respondents’ sensory acceptance of food products such as brie cheese [50], ham [51], honey [52], and extra-virgin olive oil [53]. This effect could be due to product-origin information being viewed as a cognitive cue and informational stimulus that could increase the target product’s overall acceptance [54]. Caporale and Monteleone [55] also found that, more than just the intrinsic properties of the product, there is an expectation effect on consumer acceptability generated by origin information. Caporale et al. [56] showed that origin information about olive oil samples affected product acceptability, moving consumer-liking scores toward their expectation as a consequence of an assimilation effect. An assimilation effect can also occur if liking after exposure to the product matches the expected liking based on information previously provided [57,58]. Once consumer expectations are matched, consumer satisfaction may occur and liking ratings move towards such expectations [58]. In a recent study by Kwak et al. [59], participants valued the country-of-origin of domestic wheat flour only when the bread samples made from such flour were highly acceptable in terms of sensory aspects.

### 4.2. Effect of Consumer Attitudes Toward Country-of-Origin or State-of-Origin Information on the Geographical Indication Information-Induced Variation in Consumer Acceptance of Cooked Aromatic Rice Samples

Our findings suggest that consumer attitudes toward geographical origin information can play an important role in modulating the effect of GI information on consumer acceptance of cooked aromatic rice. Some researchers have found that the impact of GI information on consumer acceptance depends on previous consumer knowledge of the product in question [57,60,61,62,63]. Schaeffer [64] found that, if both intrinsic and extrinsic product cues are available, more knowledgeable consumers will rely on intrinsic attribute information, while less knowledgeable consumers may lack the expertise to do so [64]. However, in situations where only extrinsic attributes are available as product information, more knowledgeable consumers are better able, and thus more likely, to use location of origin as a cue [56,63,64]. Similarly, Mueller and Szolnoki [65] proposed that inexperienced consumers may differ in their information processing strategies as well as in their responsiveness to intrinsic and extrinsic characteristics. This can be seen as an indication that they have not yet built strong preferences for extrinsic attributes and lack the experience to use extrinsic cues as useful predictors for how much they will like a product [65].

The impact of GI information on consumer acceptance of cooked aromatic rice was observed to a greater extent in the group of consumers that gave higher value to the “state-of-origin”, as evidenced by higher hedonic ratings in this group when GI information was presented (Table 1). Interestingly, this effect was more pronounced in the cooked rice samples of the ARV than in other varieties. If it is noted that all participants in this study were Arkansas residents, this result is quite understandable. Because Arkansas residents might tend to give higher values to local foods, they rated cooked rice of the ARV as more acceptable. Using a choice experiment, Mugera et al. [66] showed that consumers’ higher preference for local foods was related to the local attributes associated with high quality products more than to the fact that the products were locally produced. Because Arkansas is the leading rice-producing state in the U.S., participants (Arkansas residents) might have considered the ARV as having a higher sensory quality. The region-of-origin cue has been found to have a direct effect, along with an indirect effect via perceived quality, on preference for regional products in some consumer segments, especially those residents in the product’s region of origin [55,67]. When consumers are aware of the region, the GI cue facilitates consumers to associate with the region, affecting consumer evaluation on the product [55,67].

Notably, participants who gave lower values to “country-of-origin” of cooked rice samples rated cooked rice of the ARV (i.e., local food) more acceptable in the presence of GI information (Table 1). In other words, as participants gave higher values to “country-of-origin”, they rated cooked rice of the ARV as less acceptable. This result can be explained by two perspectives. First, some participants considered imported aromatic rice products (e.g., from Thailand) to have a better quality, and thus liked the cooked domestic rice variety less (e.g., ARV) when the GI information was given. Second, participants who gave higher values to domestic rice products might not be satisfied with the sensory quality of a cooked domestic rice variety, thereby decreasing hedonic ratings of such cooked rice. More specifically, a contrast effect occurred because there was a disparity between the expectation and the subsequent experience of the cooked aromatic rice sample [63,68,69]. These perspectives can also be applied to a negative correlation between COO scores and aroma liking ratings in the cooked rice sample of the imported Jasmine rice from Thailand (THV) (Table 1).

### 4.3. Effect of Geographical Indication Information on the Sensory Perception of Aromatic Rice Samples

Using the just-about-right (JAR) scale, we compared the deviations from ideal levels for specific attributes of cooked aromatic rice samples as a function of geographical indication information. In other words, we wanted to determine whether using GI information can reduce the absolute deviations from ideal levels (JAR) with respect to attribute intensity of cooked aromatic rice samples [70]. This study showed that flavor or sweetness intensities were heightened by the inclusion of GI information, bringing them closer to the just-about-right intensity (Figure 7). This result suggests that GI information can affect product perception scores toward stated expectations (or ideal levels). Similarly, Caporale et al. [56] found that, when information about the olive cultivar in olive oil was present on the label, consumer expectations for bitterness and pungency were affected. The state-of-origin of wine (California or North Dakota) also influenced wine sensory ratings and the amount of wine intake [71]. Klöckner et al. [72] proposed origin labeling as a means for helping consumers in discriminating taste differences between food products. However, as previously highlighted, such differentiations rely mostly on a selected group of consumers sufficiently informed with COO and SOO information. Klöckner et al. [72] stated that it should be assumed that knowledge and relevance of COO are considerably lower for conventional inexperienced food shoppers because they can be considered to be less involved. It thus seems reasonable to focus on marketing and educational activities that target the aromatic-rice consumer segment by increasing knowledge about rice products.

### 4.4. Implications

The results of this study provide a better understanding about how COO and SOO information can modulate perception and acceptance of cooked aromatic rice. These findings can be beneficial for U.S rice breeders and researchers as they increase their efforts to introduce domestic aromatic cultivars into the U.S market. These findings can also help the food industry better understand how geographical indication labeling can impact consumer expectations and sensory perception of food. Further research, however, should be oriented toward determining the various contextual factors that modulate the effects of COO and SOO on food acceptance, e.g., place of origin, cultural background, eating habits, upbringing, or demographic profiles (age, gender, and education) [51,52,73]. More specifically, it would be interesting to examine how the effect of GI information on consumer perception and acceptance of cooked aromatic rice samples can vary with a wider range of places of origin of aromatic rice exports to the U.S. (e.g., India, Pakistan, Vietnam, China, or Spain) [30]. Because a strong association between aromatic Jasmine rice and Thailand [74] might be related to the positive effect of the GI information on consumer acceptance of cooked aromatic rice samples, it is worth investigating whether such a positive effect of GI information is also shown when presented with GI information of other aromatic-rice exports to the U.S.

It would also be interesting to examine whether consumers’ different knowledge levels about rice products could modulate the impact of origin information on their acceptance of such a product. Feldmann and Hamm [46] reported that one of the major barriers U.S. consumers face when they have intention to support local food products is unavailability and lack of knowledge about the product origin. An increase in local rice preferences might thus not be entirely dependent on consumer demand alone, but also on company and government attempts to strengthen their local economies by adopting marketing strategies for their local products [46]. According to Conner et al. [75], products that benefit from the “locally-grown” attribute are commonly those with a product differentiation marketing strategy rather than the high-volume low-cost strategy employed by most commodity farmers in the marketplace. A few examples of this type of regional-focused marketing within the U.S. are the “Wisconsin Cheese” and the “Florida Oranges” campaigns that promote products locally-produced within the states of Wisconsin and Florida, respectively.

Some 14 aromatic rice cultivars have been developed in the public sector for production in the southern United States. Although these cultivars have met with varying degrees of success, none have received acceptance within the U.S. market sufficient to supplant Jasmine and Basmati imports [76]. Previous research using either questionnaire-based surveys or focus-group interviews has shown that consumers associate imported Jasmine rice from Thailand with having higher quality attributes compared to rice produced in other countries [74]. However, our findings showed no significant difference between imported and domestic aromatic rice samples with respect to overall liking of cooked rice samples (Figure 2), while participants liked aromas of cooked rice prepared using the imported Jasmine rice from Thailand (THV) more than cooked-rice aromas of the two domestic varieties: ARV and LAV (see also [77] and Table S2). However, since Asian consumers, a major population of aromatic-rice consumers, did not participate in this study, further studies should be conducted to test whether they also show similar patterns of acceptance toward cooked rice samples prepared using domestic and imported rice varieties, respectively [78,79,80].

## 5. Conclusions

This study provides empirical evidence that geographical indication information modulates consumer acceptance and sensory perception of cooked aromatic rice. The presence of GI information increased the acceptance of cooked aromatic rice across all the evaluated products. However, further studies should be conducted to determine whether the GI information-induced increase in consumer acceptance of cooked aromatic rice samples also exhibits in a wider range of (1) place of origin of aromatic rice exports to the U.S. and (2) consumer participant profiles. Interestingly, the presence of GI information did affect consumers differently with respect to individual attitudes toward origin-information labeling. For example, consumers who reported giving higher importance to state-of-origin information (or lower importance to country-of-origin information) in cooked aromatic rice gave higher hedonic ratings when GI information was given. In contrast, such relationships were not observed in the absence of GI information. GI information was also found to affect consumer perception of cooked aromatic rice samples with respect to JAR ratings of attributes. Flavor and sweetness intensities were closer to the JAR intensity when the GI information was given compared to when no GI information was presented. Future studies should focus on whether contextual factors such as upbringing, culture, availability, and convenience might also influence consumer attitudes and behaviors related to purchase of locally-grown aromatic rice. Governments and local entities should also assess the impact that state-focused marketing campaigns promoting U.S. grown aromatic rice could have on consumer acceptance of new aromatic native varieties.

## Figures and Tables

**Table 1:** Partial correlation coefficients (*p*-value) between the importance levels of either “country-of-origin” (COO) or “state-of-origin” (SOO) scores and hedonic ratings of three aromatic rice samples with respect to GI information condition.

Rice Variety	Attribute	COO Score ^1^		SOO Score ^2^	
		Without GI Information	With GI Information	Without GI Information	With GI Information
ARV (ARoma 17)	Overall liking	0.06 (0.57)	−0.21 (0.04)	−0.06 (0.54)	0.23 (0.02)
	Appearance liking	−0.07 (0.50)	−0.23 (0.02)	0.09 (0.37)	0.25 (0.01)
	Aroma liking	0.01 (0.92)	−0.11 (0.27)	0.01 (0.91)	0.18 (0.08)
	Flavor liking	0.01 (0.89)	−0.23 (0.02)	−0.03 (0.81)	0.25 (0.01)
	Texture liking	0.07 (0.48)	−0.20 (0.05)	−0.05 (0.64)	0.25 (0.01)
LAV (Jazzman-2)	Overall liking	−0.04 (0.67)	−0.13 (0.20)	0.01 (0.95)	0.11 (0.29)
	Appearance liking	−0.10 (0.34)	−0.19 (0.06)	0.07 (0.47)	0.15 (0.15)
	Aroma liking	−0.17 (0.10)	−0.13 (0.21)	0.18 (0.07)	0.12 (0.23)
	Flavor liking	−0.08 (0.43)	−0.15 (0.14)	0.11 (0.28)	0.14 (0.17)
	Texture liking	−0.09 (0.36)	−0.24 (0.02)	0.05 (0.66)	0.24 (0.02)
THV (Jasmine)	Overall liking	−0.05 (0.63)	−0.13 (0.19)	0.12 (0.24)	0.19 (0.06)
	Appearance liking	−0.09 (0.40)	−0.16 (0.11)	0.14 (0.16)	0.19 (0.06)
	Aroma liking	−0.03 (0.74)	−0.20 (0.049)	0.05 (0.63)	0.27 (0.008)
	Flavor liking	−0.06 (0.54)	−0.13 (0.20)	0.09 (0.40)	0.18 (0.08)
	Texture liking	−0.10 (0.34)	−0.11 (0.26)	0.20 (0.05)	0.19 (0.06)

^1^ COO score: Participants rated how important country-of-origin information was to them when consuming aromatic rice on a 9-point scale ranging from 1 (extremely unimportant) to 9 (extremely important). ^2^ SOO score: Participants rated how important state-of-origin information was to them when consuming aromatic rice on a 9-point scale ranging from 1 (extremely unimportant) to 9 (extremely important).

**Table 2:** Comparisons between the two geographical indication (GI) information conditions with respect to percentage of Just-About-Right (JAR) for each attribute in each rice variety.

Rice Variety	Attribute	GI Information Condition		*Χ*^2^-Value (*p*-Value)
		Without GI Information	With GI Information	
ARV (ARoma 17)	Color	82 (82.8%)	83 (83.8%)	0.04 (0.85)
	Aroma	63 (63.6%)	66 (66.7%)	0.20 (0.65)
	Flavor	47 (47.5%)	56 (56.6%)	1.64 (0.20)
	Saltiness	34 (34.3%)	42 (42.4%)	1.37 (0.24)
	Sweetness	53 (53.5%)	55 (55.6%)	0.08 (0.78)
	Bitterness	72 (72.7%)	75 (75.8%)	0.24 (0.63)
	Firmness	59 (59.6%)	61 (61.6%)	0.08 (0.77)
	Stickiness	43 (43.4%)	43 (43.4%)	0.00 (1.00)
	Chewiness	58 (58.6%)	64 (64.7%)	0.77 (0.38)
LAV (Jazzman-2)	Color	61 (61.6%)	80 (80.8%)	8.89 (0.003)
	Aroma	64 (64.7%)	66 (66.7%)	0.09 (0.76)
	Flavor	47 (47.5%)	59 (59.6%)	2.92 (0.09)
	Saltiness	44 (44.4%)	44 (44.4%)	0.00 (1.00)
	Sweetness	45 (45.5%)	59 (59.6%)	3.97 (0.046)
	Bitterness	61 (61.6%)	68 (68.7%)	1.09 (0.30)
	Firmness	78 (78.8%)	79 (79.8%)	0.03 (0.86)
	Stickiness	66 (66.7%)	72 (72.7%)	0.86 (0.43)
	Chewiness	72 (72.7%)	71 (71.7%)	0.03 (0.87)
THV (Jasmine)	Color	88 (88.9%)	85 (85.9%)	0.41 (0.52)
	Aroma	79 (79.8%)	84 (84.9%)	0.87 (0.35)
	Flavor	58 (58.6%)	61 (61.6%)	0.19 (0.66)
	Saltiness	45 (45.5%)	44 (44.4%)	0.02 (0.89)
	Sweetness	58 (58.6%)	55 (55.6%)	0.19 (0.67)
	Bitterness	73 (73.7%)	76 (76.8%)	0.24 (0.62)
	Firmness	59 (59.6%)	66 (66.7%)	1.06 (0.30)
	Stickiness	50 (50.5%)	47 (47.5%)	0.18 (0.67)
	Chewiness	62 (62.6%)	70 (70.7%)	1.45 (0.23)

## Supplementary Materials

The following are available online at https://www.mdpi.com/2304-8158/9/12/1843/s1: Table S1. Physicochemical properties of the uncooked aromatic rice samples used in this study, Table S2. Sensory attributes of the three cooked aromatic rice samples evaluated by five trained panelists in the descriptive sensory analysis, Table S3. Terms, definitions, and reference intensities used in descriptive sensory analysis of cooked aromatic rice samples, Table S4. A list of terms that have been reported by at least 10 out of 99 consumer participants with respect to what they liked for the three cooked aromatic rice samples, and Table S5. A list of terms that have been reported by at least 10 out of 99 consumer participants with respect to what they disliked for the three cooked aromatic rice samples.
