# Supplementary material for: Effect of Geographical Indication Information on Consumer Acceptability of Cooked Aromatic Rice
Source: Foods. 2020 Dec 11;9(12):1843. doi: 10.3390/foods9121843 (PMC7763253; doi:10.3390/foods9121843)
Supplement: Supplementary file 1 [file foods-09-01843-s001.pdf]

Table S1. Physicochemical properties of the uncooked aromatic rice samples used in this study

Physicochemical properties	ARoma 17 (ARV)	Jazzman-2 (LAV)	Jasmine (THV)	*P*-value
Amylose (%, db.)	12.57[a] (± 0.3)[1]	13.18[a] (± 0.10)	11.20[b] (± 0.0)	0.003
Crude protein (%)	7.74[a] (± 0.05)	7.53[b] (± 0.07)	7.26[c] (± 0.06)	< 0.001
Surface lipid content (%)[2]	0.57[b] (± 0.01)	0.66[a] (± 0.02)	0.38[c] (± 0.01)	< 0.001
Pasting temperature (°c)	87.07[a] (± 0.09)	87.07[a] (± 0.05)	76.07[b] (± 0.05)	< 0.001
Peak viscosity (cP)	3,428.67[b] (± 72.6)	3,040.34[c] (± 61.16)	4,165.00[a] (± 73.96)	< 0.001
Final viscosity (cP)	3,006.00[b] (± 22.69)	3,657[a] (± 27.28)	3,063.34[b] (± 25.96)	< 0.001
Setback (cP)	-422.67[b] (± 88.13)	616.67[a] (± 84.31)	11,001.67[c] (± 62.94)	< 0.001
L*	69.43[b] (± 0.18)	67.24[c] (± 0.28)	72.78[a] (± 0.36)	< 0.001
a*	0.63[a] (± 0.29)	0.94[a] (± 0.20)	-0.82[b] (± 0.24)	0.001
b*	17.53[b] (± 0.17)	19.25[a] (± 0.32)	17.60[b] (± 0.34)	0.001

[1]Values represent the mean (± standard deviation) of triplicate measurements. Different superscript letters within a row indicate a significant difference ($P < 0.05$) determined by *post hoc* test using Tukey's Honest Significant Difference.

[2]Surface lipid content was measured by NIR spectroscopy (NIR-DA 7200, Perten Instruments, Huddinge, Sweden).

Table S2. Sensory attributes of the three cooked rice samples evaluated by five trained panelists in the descriptive sensory analysis

Attributes	Cooked aromatic rice Samples			*F*-value (*P*-value)					
	ARoma 17 (ARV)	Jazzman-2 (LAV)	Jasmine (THV)	Sample (S)	Assessor (A)	Repetition (R)	S × A	A × R	S × R
**Appearance**									
Degree of Whiteness	9.90^{b} (± 1.57)[1]	9.30^{b} (± 2.01)	10.84^{a} (± 1.08)	29.98 (< 0.001)	76.32 (< 0.001)	1.60 (0.23)	4.38 (0.01)	1.80 (0.15)	1.99 (0.15)
Glossiness	8.77^{a} (± 2.14)	3.98^{b} (± 1.06)	9.81^{a} (± 2.16)	103.46 (< 0.001)	12.71 (< 0.001)	0.63 (0.54)	4.35 (0.01)	0.38 (0.92)	1.00 (0.44)
Grain Integrity	4.29^{a} (± 1.50)	4.38^{a} (± 1.16)	4.44^{a} (± 1.48)	0.05 (0.95)	2.41 (0.09)	0.30 (0.75)	1.69 (0.18)	0.98 (0.48)	0.88 (0.50)
Color Uniformity	11.8^{b} (± 1.75)	10.46^{c} (± 1.68)	13.38^{a} (± 0.77)	50.40 (< 0.001)	24.80 (< 0.001)	0.60 (0.56)	2.16 (0.09)	2.09 (0.10)	0.38 (0.82)
**Aroma**									
Sulfur	6.96^{a} (± 3.24)	6.69^{a} (± 3.09)	4.16^{b} (± 3.53)	8.62 (< 0.001)	14.78 (< 0.001)	0.91 (0.42)	2.16 (0.09)	1.02 (0.46)	1.67 (0.21)
Floral	3.04^{a} (± 1.60)	4.35^{a} (± 1.92)	4.48^{a} (± 1.81)	2.82 (0.09)	1.92 (0.16)	0.48 (0.63)	1.03 (0.46)	0.40 (0.90)	0.76 (0.56)
Starchy	7.42^{a} (± 2.05)	7.08^{a} (± 3.17)	8.88^{a} (± 1.84)	2.75 (0.09)	3.17 (0.04)	0.88 (0.43)	0.86 (0.57)	1.36 (0.29)	0.25 (0.91)
Grainy	7.56^{b} (± 3.07)	7.99^{b} (± 2.83)	9.47^{a} (± 1.66)	4.76 (0.02)	8.44 (< 0.001)	0.12 (0.89)	0.60 (0.76)	2.96 (0.03)	2.50 (0.08)
Feedy	3.60^{a} (± 2.93)	4.13^{a} (± 2.92)	3.63^{a} (± 2.21)	0.70 (0.51)	22.00 (< 0.001)	3.85 (0.04)	1.86 (0.14)	3.53 (0.02)	2.18 (0.12)
Nutty	5.34^{b} (± 3.12)	5.06^{b} (± 3.17)	7.29^{a} (± 2.93)	6.59 (0.01)	17.85 (< 0.001)	0.77 (0.48)	1.17 (0.37)	2.21 (0.09)	0.43 (0.79)
**Flavor**									
Sulfur	3.88^{a} (± 1.73)	3.76^{a} (± 1.44)	3.60^{a} (± 1.60)	0.16 (0.85)	6.14 (< 0.001)	1.21 (0.32)	0.46 (0.87)	1.24 (0.34)	10.00 (0.60)
Floral	2.43^{a} (± 1.53)	2.71^{a} (± 1.59)	3.42^{a} (± 2.00)	1.27 (0.31)	1.11 (0.39)	1.49 (0.26)	1.09 (0.42)	1.04 (0.45)	0.56 (0. 69)
Sweet Aromatics	2.32^{a} (± 0.90)	1.73^{a} (± 0.83)	2.66^{a} (± 1.64)	1.88 (0.19)	2.12 (0.13)	0.82 (0.46)	0.45 (0.87)	0.58 (0.78)	0.15 (0.96)
Starchy	9.71^{a} (± 1.80)	9.15^{a} (± 2.95)	9.82^{a} (± 1.78)	0.57 (0.58)	2.21 (0.11)	1.09 (0.36)	1.06 (0.43)	3.72 (0.01)	0.30 (0.87)
Grainy	6.58^{a} (± 2.80)	6.76^{a} (± 3.11)	5.57^{a} (± 2.53)	1.10 (0.36)	4.90 (0.01)	0.48 (0.63)	1.69 (0.18)	1.60 (0.20)	0.28 (0.89)
Feedy	1.91^{ab} (± 1.45)	1.94^{a} (± 1.48)	0.89^{b} (± 0.72)	5.08 (0.02)	3.74 (0.03)	0.76 (0.49)	1.18 (0.37)	1.72 (0.17)	2.97 (0.05)
Wet Cardboard	3.44^{a} (± 2.29)	3.96^{a} (± 2.81)	3.78^{a} (± 1.62)	0.38 (0.69)	5.14 (0.01)	5.37 (0.02)	0.91 (0.53)	2.47 (0.06)	2.26 (0.11)

Nutty	4.50^{a} (± 2.39)	4.70^{a} (± 2.27)	4.32^{a} (± 2.15)	0.24 (0.79)	14.25 (< 0.001)	4.34 (0.03)	1.29 (0.32)	0.93 (0.52)	0.58 (0.68)
Earthy/Dirty	0.43^{a} (± 0.38)	0.84^{a} (± 0.71)	0.76^{a} (± 1.00)	3.22 (0.07)	8.27 (< 0.001)	3.36 (0.06)	2.76 (0.04)	3.26 (0.02)	2.04 (0.14)
Metallic	1.83^{a} (± 1.22)	2.22^{a} (± 1.18)	2.14^{a} (± 1.60)	0.57 (0.58)	6.26 (< 0.001)	0.90 (0.43)	1.32 (0.30)	2.08 (0.10)	0.68 (0.62)
**Basic tastes**									
Sweet taste	0.78^{a} (± 0.50)	0.69^{a} (± 0.37)	0.86^{a} (± 0.53)	1.51 (0.25)	14.60 (< 0.001)	1.07 (0.37)	3.09 (0.03)	2.75 (0.04)	1.82 (0.17)
Bitter taste	0.84^{a} (± 0.77)	1.36^{a} (± 1.43)	0.66^{a} (± 0.88)	3.46 (0.06)	10.25 (< 0.001)	0.39 (0.68)	2.95 (0.03)	0.59 (0.77)	0.88 (0.50)
Salty taste	0.86^{a} (± 0.90)	0.75^{a} (± 0.62)	0.90^{a} (± 0.67)	0.51 (0.61)	20.26 (< 0.001)	0.92 (0.42)	1.32 (0.30)	2.80 (0.04)	2.20 (0.12)
**Texture**									
Hardness	3.39^{b} (± 0.60)	4.23^{a} (± 0.66)	2.86^{b} (± 0.87)	16.48 (< 0.001)	2.97 (0.05)	0.58 (0.57)	2.17 (0.09)	0.61 (0.76)	0.24 (0.91)
Cohesiveness of Mass	8.03^{a} (± 2.37)	6.44^{a} (± 2.96)	8.26^{a} (± 3.03)	4.16 (0.04)	12.72 (< 0.001)	1.82 (0.19)	1.18 (0.37)	1.91 (0.13)	0.94 (0.46)
Toothpull	4.32^{a} (± 1.49)	3.36^{a} (± 1.37)	3.78^{a} (± 1.29)	3.95 (0.04)	10.48 (< 0.001)	2.76 (0.09)	1.55 (0.22)	1.93 (0.13)	1.21 (0.35)
Toothpack	4.70^{a} (± 2.27)	5.17^{a} (± 1.97)	4.42^{a} (± 2.30)	0.69 (0.52)	7.49 (< 0.001)	0.79 (0.47)	0.81 (0.60)	1.63 (0.19)	0.41 (0.80)
Mouthcoat	7.76^{a} (± 1.79)	6.22^{a} (± 2.86)	6.55^{a} (± 2.36)	2.83 (0.09)	2.42 (0.09)	1.29 (0.30)	2.24 (0.08)	3.02 (0.03)	0.60 (0.67)
Loose Particles	5.10^{a} (± 2.01)	5.50^{a} (± 2.09)	5.64^{a} (± 1.89)	0.48 (0.63)	4.45 (0.01)	0.02 (0.98)	2.76 (0.04)	1.65 (0.19)	0.72 (0.59)
Manual Stickiness	11.16^{a} (± 1.00)	10.67^{a} (± 1.71)	11.47^{a} (± 1.07)	4.86 (0.02)	22.56 (< 0.001)	1.06 (0.37)	2.10 (0.10)	2.43 (0.06)	1.41 (0.28)

[1]Values represent the mean (± standard deviation) of triplicate measurements. Different superscript letters within a row indicate a significant difference ($P < 0.05$) determined by *post hoc* test using Tukey's Honest Significant Difference (HSD).

Table S3. Terms, definitions, and reference intensities used in descriptive sensory analysis of cooked aromatic rice samples

Terms	Definitions	References[1]
Appearance		
Degree of Whiteness	The degree to which the sample is pure white.	Ref A: 3.0<br>Ref C: 13.0
Glossiness	The degree to which the kernel shines.	Ref C: 6.0
Grain Integrity	The degree to which the kernels are cracked or split apart.	Ref B: 12.0
Color Uniformity	The degree to which the rice sample is uniform in color.	Ref C: 13.0
Aroma		
Sulfur	The aroma associated with struck matches and boiled eggs.	Ref G: 2.0<br>Ref F: 8.0
Floral	The aroma associated with a non-specific floral note and sometimes described as minty.	Ref F: 4.0
Starchy	The aroma associated with the starch of a particular grain source.	Ref G: 6.0<br>Ref D: 10.0
Grainy	A general term used to describe the aroma of raw or cooked grains and popcorn	Ref F: 7.0<br>Ref E: 12.0
Feedy	The aroma associated with a mixture of grains reminiscent of animal feed.	Ref E: 6.0
Nutty	The aroma associated with nuts or nutmeats that cannot be tied to a specific origin.	Ref E: 12.0
Flavor		
Sulfur	The aromatic associated with struck matches and boiled eggs.	Ref G: 2.0<br>Ref F: 8.0
Floral	The aromatic associated with a non-specific floral note and sometimes described as minty.	Ref F: 4.0
Sweet Aromatics	Aromatics associated to sweet tasting products but not ‘basic taste sweet’	Ref E: 5.0
Starchy	The aromatic associated with the starch of a particular grain source.	Ref G: 6.0<br>Ref D: 10.0
Grainy	A general term used to describe the aromatics of raw or cooked grains and popcorn	Ref F: 7.0<br>Ref E: 12.0
Feedy	The aromatic associated with a mixture of grains reminiscent of animal feed.	Ref E: 6.0
Wet Cardboard	The aromatic associated with early stages of oxidation.	Ref G: 3.0<br>Ref D: 7.0
Nutty	The aromatic associated with nuts or nutmeats that cannot be tied to a specific origin.	Ref E: 12.0
Earthy/Dirty	Aromatic associated with damp earth aromas.	Ref D: 3.0

Metallic	Aromatic associated with metals, tinny or irony.	Ref B: 3.0
Basic Taste		
Sweet	The basic taste, perceived on the tongue, stimulated by sugars and high potency sweeteners.	Sucrose solution 2% (v/v): 2.0<br>5% (v/v): 5.0
Bitter	The basic taste, perceived on the tongue, stimulated by substances such as quinine, caffeine, and certain other alkaloids.	Caffeine solution<br>0.05% (v/v): 2.0<br>0.08% (v/v): 5.0
Salt	The basic taste, perceived on the tongue, stimulated by sodium salt, especially sodium chloride.	NaCl solution<br>0.20% (v/v): 2.0<br>0.35% (v/v): 5.0
Texture		
Hardness	The force required to compress the sample.	Ref D: 2.0<br>Ref B: 6.0<br>Ref C: 10.0
Cohesiveness of Mass	The amount the chewed sample holds together.	Ref C: 3.0<br>Ref A: 7.0<br>Ref D: 12.0
Toothpull	The force required to separate the teeth during mastication.	Ref A: 6.0
Toothpack	The amount of product packed into the crowns of your teeth after mastication.	Ref B: 3.0<br>Ref C: 8.0
Mouthcoat	The amount and degree of residue felt by the tongue when moved over the surface of the mouth.	Ref D: 4.0<br>Ref C: 13.0
Loose Particles	The amount of particles remaining in and on the surface of the mouth after swallowing.	Ref D: 4.0<br>Ref C: 13.0
Manual Stickiness	The force required to separate the fingers after compressing the sample between the thumb and forefinger.	Ref B: 2.0<br>Ref D: 10.0

[1]Reference A: 30 g Riceland Brown Rice (water: rice ratio = 3.5:1; cooking time = 35 min) (Riceland Foods, Stuttgart, AR, USA)

Reference B: 30 g Uncle Ben's Converted Brown Rice (water: rice ratio = 3.5:1; cooking time = 25 min) (Mars Food, McLean, VA, USA)

Reference C: 30 g Riceland Brown Rice (water: rice ratio = 3.5:1; cooking time = 25 min) (Riceland Foods, Stuttgart, AR, USA)

Reference D: 30 g Riceland Extra Long- Grain Rice (water: rice ratio = 3.5:1; cooking time = 25 min) (Riceland Foods, Stuttgart, AR, USA)

Reference E: 30 g Golden Star Jasmine Brown Rice (water: rice ratio = 2.5:1; cooking time = 35 min) (Golden Star Trading, Torrance, CA, USA)

Reference F: Golden Star Jasmine Rice (water: rice ratio = 2:1; cooking time = 25 min) (Golden Star Trading, Torrance, CA, USA)

Reference G: 30 g Mahatma Jasmine Rice (water: rice ratio = 2:1; cooking time = 25 min) (Riviana Foods, Houston, TX, USA).

Table S4. A list of terms that have been reported by at least 10 out of 99 consumer participants with respect to what they liked for the three cooked aromatic rice samples in the two geographical indication (GI) information conditions

(*N* = 99)

Rice variety	GI information condition	Terms	Frequency
ARoma 17 (ARV)	No information	Flavor	29
		Texture	21
		Aroma	18
		Color	15
		Appearance	13
		Stickiness	12
		Taste	11
		Chewiness	10
	GI information	Flavor	31
		Texture	22
		Aroma	14
		Color	14
		Appearance	11
		Chewiness	10
		Taste	10
Jazz-2 (LAV)	No information	Texture	28
		Flavor	23
		Aroma	17
		Chewiness	14
		Firmness	12
		Stickiness	12
		Taste	11
	GI information	Texture	35
		Flavor	27
		Appearance	15
		Aroma	13
		Stickiness	13
		Taste	13
		Firmness	12
Jasmine (THV)	No information	Flavor	28
		Aroma	21
		Texture	19
		Appearance	17
		Color	14
		Stickiness	13
		Taste	12
	GI information	Flavor	30
		Texture	25

Aroma	24
Appearance	17
Color	13
Stickiness	11

Table S5. A list of terms that have been reported by at least 9 out of 99 consumer participants with respect to what they disliked for the three cooked aromatic rice samples in the two geographical indication (GI) information conditions

(*N* = 99)

Rice variety	GI information condition	Terms	Frequency
ARoma 17 (ARV)	No information	Stickiness	32
		Flavor	19
		Bland flavor	14
		Saltiness	14
		Chewiness	13
	GI information	Stickiness	38
		Flavor	23
		Chewiness	16
		Saltiness	15
Jazz-2 (LAV)	No information	Flavor	20
		Bitterness	18
		Color	17
		Dry	14
		Taste	13
		Aftertaste	10
		Bland flavor	10
	GI information	Flavor	20
		Bitterness	14
		Dry	14
		Color	14
		Chewiness	13
		Saltiness	13
		Stickiness	13
		Bland flavor	11
		Appearance	11
Jasmine (THV)	No information	Stickiness	29
		Flavor	24
		Bland flavor	12
		Chewiness	12
	GI information	Stickiness	46
		Flavor	15
		Chewiness	12
		Saltiness	12
		Aroma	10
		Bland flavor	10
